# High-frequency marker haplotypes in the genomic selection of dairy cattle

**DOI:** 10.1007/s13353-019-00489-9

**Published:** 2019-03-15

**Authors:** Anna Mucha, Heliodor Wierzbicki, Stanisław Kamiński, Kamil Oleński, Dorota Hering

**Affiliations:** 1 Department of Genetics, University of Environmental and Life Sciences, Kożuchowska 7, 51-613 Wroclaw, Poland; 2 Department of Animal Genetics, University of Warmia and Mazury, Oczapowskiego 5, 10-718 Olsztyn, Poland

**Keywords:** Genomic evaluation, Haplotype, Linear model, Single-nucleotide polymorphism

## Abstract

The aim of this study was to predict the genomic breeding value (DGV) of production, selected conformation and reproductive traits, and somatic cell score of dairy cattle in Poland using high-frequency marker haplotypes. The dataset consisted of phenotypic, genotypic, and pedigree data of 1216 Polish Holstein-Friesian bulls. The genotypic data consisted of 54,000 single-nucleotide polymorphisms (SNPs). The data were divided into two subsets: a test dataset (*n* = 1064) and a validation dataset (*n* = 152). Genotypic data were selected using three criteria: the percentage of missing genotypes, minor allele frequency, and linkage disequilibrium. The purpose of the data selection was to identify blocks of SNPs that were then used for the construction of haplotypes. Only haplotypes with a frequency higher than 25% were selected. DGV was predicted using four variants of a linear model with random haplotype effects and deregressed breeding values as the response variables. The accuracy of genomic prediction was checked by comparing DGVs with estimated breeding values (EBVs) using two methods: Pearson’s correlations and the regression of EBV on DGV. The use of high-frequency haplotypes showed a tendency to underestimate DGVs. None of the models tested was clearly superior with regard to the traits studied. DGVs of production and conformation traits as well as somatic cell score (medium or high heritability traits) were more accurate than those estimated for fertility traits (low heritability traits).

## Introduction

The genomic selection introduced by Meuwissen et al. (2001) has received much attention in animal breeding, because it provides predictions of the breeding values at a young age of animals with higher accuracy than breeding values based on parent average (Hess et al. 2017; Van Raden 2008). The potential benefits of using genomic selection were outlined by Schaeffer (2006). He showed that the genetic progress of a selected population of Canadian Holstein cattle would be doubled compared to the selection based on offspring phenotypes. He also stated that the use of genomic selection would reduce financial costs by 92% compared to traditional selection. Other benefits of genomic selection are decreased generation interval and the possibility of identifying recessive lethals (Wiggans et al. 2017). Furthermore, due to the lower production cost, a much larger number of bulls can be selected, which leads to a better management of genetic resources and limitation of inbreeding trends (Boichard et al. 2016).

The genomic breeding values (GBVs) in dairy cattle are usually predicted using single SNPs. However, a haplotype approach to the prediction of genomic breeding values using high density data is an alternative to single-marker methods (Calus et al. 2008; Cuyabano et al. 2014; Jónás et al. 2016; Hess et al. 2017). Building haplotypes based on linkage disequilibrium reduces the number of variables without the loss of information (Cuyabano et al. 2014).

An important benefit of haplotypes over SNP markers is their superior ability to identify mutations (Cuyabano et al. 2014). In addition, the use of haplotypes in genomic selection is advantageous because this approach treats the haplotype as a functional unit that contains the combined effects of tightly linked cis-acting causal variants (Da 2015; Garnier et al. 2013). On the other hand, the main disadvantage of models with marker haplotypes is that the number of effects to be estimated is significantly larger than that for SNP models (Calus et al. 2008). There are over a million haplotype alleles for a block of 20 biallelic SNPs, many of which occur at a low frequency (Hess et al. 2017). However, the number of haplotype effects to be estimated can be reduced by including more SNPs per haplotype or by using only haplotypes with a high frequency in the population. Discarding rare haplotype alleles also reduces computation time with little expected decrease in prediction accuracy (Gianola 2013). Other effective methods that reduce the number of explanatory variables in the linear model are, for example, the use of linkage disequilibrium (LD) to determine where a haplotype starts and ends in the genome (Gabriel et al. 2002), or the definition of haplotypes by setting windows with a fixed number of SNPs to form a haplotype (Villumsen et al. 2008).

The main purpose of this study was to predict the genomic breeding values of the production, selected conformation and reproductive traits, as well as somatic cell scores of Polish Holstein-Friesian cattle using high-frequency marker haplotypes. The study was divided into three stages: (1) selection of SNPs and blocks of linked markers, (2) construction of haplotypes and estimation of their frequency in the population under scrutiny, and (3) prediction of genomic breeding values using high-frequency haplotypes and comparison of the linear models applied.

## Material and methods

### Data

**Fig. 1:**
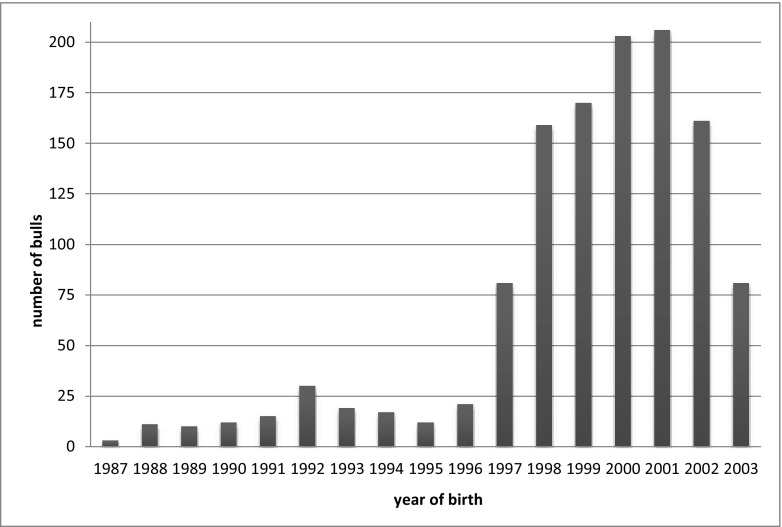
The distribution of the number of bulls analyzed with respect to the year of their birth

The dataset included the phenotype, genotype, and pedigree data of 1216 bulls of the Polish Holstein-Friesian breed. All the animals were born between 1987 and 2003. The most numerous group were born between 1997 and 2003 (*n* = 1061). The distribution of the number of bulls analyzed with respect to the year of their birth is shown in Fig. 1.

To compare prediction models most often so-called a K-fold cross-validation (K-fold CV) is used (Gianola and Schön 2016). However, in the present study, the CV layout was based on a generational partition of the dataset into two subsets (Perez-Cabal et al. 2012). One was the test dataset consisting of 1064 of the oldest individuals (87.5% of all the bulls) to train the model; the other contained the remaining 152 youngest individuals—this was the validation dataset. It has been shown (Daetwyler et al. 2008; Erbe et al. 2010) that larger predictive correlations in genomic selection are proportional to training sample size, thus, to increase predictive ability of the model, the test dataset was seven times larger than the validation dataset.

The study involved the following traits: milk yield (MY), fat yield (FY), protein yield (PY), stature (STA), overall feet and leg score (OFL), udder support (USU), non-return rate of cows (NRK), rest before pregnancy (PRP), time between pregnancies (OMC), and somatic cell score (SCS). MY, FY, and PY were measured in kilograms, while STA, OFL, and USU were included in the linear assessment expressed as points awarded (from 50 to 100 points for ST A and OFL, from 1 to 9 points for USU). NRK was 1 if a cow did not show symptoms of oestrus within 56 days of the first insemination, and 0 otherwise. PRP and OMC were expressed in days. SCS was calculated by converting the somatic cell count (SCC) using the following formula:

$$ \mathrm{SCS}={\log}_2\left(\frac{\mathrm{SCC}}{100000}\right)+3. $$

Estimated breeding values (EBVs) were obtained using a test day model with random regressions (Strabel et al. 2005). Heritabilities and variance components of the traits analyzed were estimated within the framework of the national genetic evaluation system of Polish Holstein-Friesian cattle (Interbull 2012). Their values are listed in Table 1.

**Table 1:** Heritabilities (*h*^2^) and genetic variances (*σ*^2^_*g*_) of the analyzed traits

	Trait									
	MY	FY	PY	STA	OFL	USU	NRK	PRP	OMC	SCS
*h* ^2^	0.33	0.29	0.29	0.54	0.11	0.20	0.02	0.05	0.08	0.32
${\sigma}_g^2$	213,490	330.10	181.30	5.50	0.89	0.37	0.00451	171.50	557.40	28,737

Genotypic data were obtained using Illumina BovineSNP50 BeadChip (Matukumalli et al. 2009). The use of microarrays enabled the analysis of 54,001 SNPs for each individual. Out of 54,001 SNPs, 46,267 SNPs were selected for the DGV estimation based on their minor allele frequency (≥ 1%) and call rate (≥ 90%). This set of SNPs was subjected to further selection (the SNP selection criteria are described in the next subsection).

### Statistical analysis

The statistical analysis was divided into three main steps: (1) selection of SNPs and blocks of linked markers, (2) construction of haplotypes and estimation of their frequency in the population analyzed, and (3) prediction of genomic breeding values using haplotypes and comparison of the linear models applied.

The first step in the SNP selection was to remove from the dataset those markers for which the missing data (genotypes) represented more than 10% of the total (GENO < 0.1). Then, the minor allele frequency (MAF) was used. Two limiting values were considered: 1 and 5%. As a result of this selection, two subsets of data were created: one with MAF > 0.01 and the other with MAF > 0.05. Based on the data subsets created in the previous step, blocks of linked SNPs were selected. LD of SNPs was measured using *r*^2^. Two limiting values were used: *r*^2^ ≥ 0.8 and *r*^2^ ≥ 0.9. Within each chromosome, *r*^2^ was calculated for each pair of SNPs. Linked markers were combined into blocks. As a result, four subsets of data were prepared: (1) MAF > 0.01 and *r*^2^ ≥ 0.8, (2) MAF > 0.01 and *r*^2^ ≥ 0.9, (3) MAF > 0.05 and *r*^2^ ≥ 0.8, and (4) MAF > 0.05 and *r*^2^ ≥ 0.9. SNP selection was carried out using the PLINK program (Purcell et al. 2007; Purcell 2010). Owing to the largest variety of block sizes, the first of the abovementioned subsets (MAF > 0.01 and *r*^2^ ≥ 0.8) was selected for further analysis. The blocks of SNPs were then used for the construction of haplotypes. This stage of the study, together with the estimation of haplotype frequency in the population, was done using the PHASE program (Stephens et al. 2001). In order to substantially reduce the number of explanatory variables in the linear models, only haplotypes with a frequency higher than 25% were selected for further analysis.

The genomic breeding values were predicted using four variants of the following linear model for each trait analyzed:

$$ y=\mu +\mathrm{Zh}+\varepsilon, $$

where *y* is the vector of deregressed breeding values, *μ* is the overall mean, *Z* is the design matrix for the random haplotype effects, *h* is the vector of the random haplotype effects, and *ε* is the vector of random errors, where *ε* ~ *N* (0, ${\sigma}_e^2$) and ${\sigma}_{\mathrm{e}}^2$ is the error variance. The EBVs were deregressed using the method of Jairath et al. (1998).

Two variants of the above linear model (model 1 and model 2) used genetic variance equally divided into all haplotypes (regardless of their length), where $h\sim N\left(0,\frac{\sigma_g^2}{\ \mathrm{number}\ \mathrm{of}\ \mathrm{haplotypes}}\right)$, $\mathrm{and}\ {\sigma}_g^2$ is the genetic variance of the trait. Furthermore, two types of the *Z* matrix were employed: model 1 used the *Z* matrix containing the probabilities of a given haplotype being passed on by a particular bull, while model 2 used the *Z* matrix consisting of only ones, if a given bull had a specific haplotype, and of zeros if it did not. Two other variants of the linear model (model 3 with the *Z* matrix defined as in model 1, and model 4 with the *Z* matrix defined as in model 2) used genetic variance unequally divided into all haplotypes (the larger the haplotype, the greater the part of the genetic variance assigned to it), where $h\sim N\left(0,{\sigma}_g^2\bullet \frac{\mathrm{haplotype}\ \mathrm{size}}{\mathrm{number}\ \mathrm{of}\ \mathrm{alleles}}\right)$.

The genomic breeding value of the *i*th individual was estimated as follows: DGV_*i*_ = ∑_*j*_*Z*_ij_*h*_*j*_.

The accuracy of the prediction of DGV was checked using two methods: Pearson’s correlations between EBV and DGV and the regression of EBV on DGV (Meuwissen et al. 2001).

All statistical analyses (unless otherwise stated) were performed using the R project (R Core Team 2011).

## Results

### Constructing haplotypes and estimating their frequency in the population

Haplotypes were constructed on the basis of the blocks selected—those selected from SNPs fulfilling the criteria of MAF > 0.01 and *r*^2^ ≥ 0.8 were considered the best set for haplotype construction. The reason for such a decision was the large variety of block sizes, especially the fact that the largest blocks were created in this subset. Table 2 shows the numbers of haplotypes constructed for individual chromosomes. The largest number of haplotypes was obtained for chromosome 26 (3709 haplotypes), whereas the smallest number of haplotypes was constructed for the sex chromosome (99 haplotypes) and for chromosome 28 (137 haplotypes). Also, chromosomes 23 and 24 were distinguished by the number of haplotypes, where their number exceeded 2000 (2069 and 2843, respectively). The number of haplotypes was dependent on block size. The larger the block, the more possible combinations of alleles, and thus the more possible haplotypes. The criterion for the selection of haplotypes was their frequency in the population. Only haplotypes which appeared in at least 25% of the individuals in the population were taken into account. Ultimately, the number of haplotypes was reduced from over 21,000 to 5682.

**Table 2:** The number of haplotypes constructed for each chromosome

Chromosome no.	All constructed haplotype	Number of haplotypes with freqency > 25%
1	1050	409
2	774	321
3	748	303
4	717	269
5	524	215
6	711	307
7	688	266
8	776	299
9	456	183
10	551	223
11	549	225
12	361	147
13	493	183
14	623	234
15	355	159
16	539	207
17	407	142
18	267	114
19	295	127
20	526	183
21	327	141
22	309	127
23	2069	175
24	2843	228
25	167	71
26	3709	162
27	145	66
28	137	63
29	227	94
Total	21,343	5643
30 (allosome)	99	39
Total	21,442	5682

### Predicting genomic breeding values

Tables 3 and 4 show the linear correlation coefficients between DGV and EBV for the test and validation datasets, respectively. In the test dataset, the strongest correlations were estimated for STA, FY, MY, PY, USU, and SCS, whereas lower correlations were estimated for reproductive traits (NRK, PRP, OMC). The linear correlation coefficients estimated in the validation dataset were lower; however, as in the test dataset, stronger correlations were estimated for medium and high heritability traits, while for the low heritability traits the estimated correlations were lower.

**Table 3:** Pearson’s correlation coefficients between DGV and EBV for the test dataset

Model	Trait									
	MY	FY	PY	STA	OFL	USU	NRK	PRP	OMC	SCS
1	0.72	0.75	0.71	0.79	0.67	0.71	0.64	0.63	0.63	0.70
2	0.72	0.75	0.71	0.79	0.67	0.71	0.64	0.63	0.63	0.70
3	0.72	0.75	0.71	0.79	0.66	0.71	0.63	0.63	0.63	0.71
4	0.72	0.75	0.71	0.80	0.66	0.71	0.63	0.63	0.63	0.71

**Table 4:** Pearson’s correlation coefficients between DGV and EBV for the validation dataset

Model	Trait									
	MY	FY	PY	STA	OFL	USU	NRK	PRP	OMC	SCS
1	0.45	0.33	0.41	0.29	0.38	0.43	0.34	0.24	0.38	0.39
2	0.45	0.33	0.41	0.29	0.38	0.43	0.34	0.24	0.38	0.39
3	0.47	0.36	0.42	0.31	0.37	0.42	0.33	0.24	0.38	0.39
4	0.47	0.36	0.42	0.31	0.37	0.43	0.34	0.23	0.38	0.39

Tables 5 and 6 show regression coefficients of EBV on DGV for the test and validation datasets. All the tested models underestimated genomic breeding values. In the test dataset, the regression coefficients closest to the desired value (though a few times higher) were obtained for models 3 and 4. Models 1 and 2 displayed an evident tendency to underestimate breeding values, which was manifested by regression coefficients significantly exceeding unity. This tendency was particularly pronounced for NRK, PRP, and OMC (low heritability traits), and also for OFL and USU.

**Table 5:** Regression coefficients of EBV on DGV for the test dataset

Model	Trait									
	MY	FY	PY	STA	OFL	USU	NRK	PRP	OMC	SCS
1	3.43	6.73	3.34	2.40	11.93	7.35	48.99	19.90	12.38	4.54
2	3.44	6.73	3.35	2.40	11.94	7.34	48.98	19.90	12.38	4.54
3	2.95	5.88	2.91	2.17	9.85	6.29	38.60	17.05	10.67	4.10
4	2.96	5.87	2.92	2.19	9.89	6.29	38.61	17.04	10.69	4.10

**Table 6:** Regression coefficients of EBV on DGV for the validation dataset

Model	Trait									
	MY	FY	PY	STA	OFL	USU	NRK	PRP	OMC	SCS
1	3.83	5.63	2.79	1.30	10.58	6.54	32.80	10.04	9.33	4.12
2	3.83	5.60	2.79	1.30	10.56	6.53	32.85	9.98	9.30	4.09
3	3.39	5.14	2.55	1.24	8.68	5.27	25.25	8.29	7.92	3.62
4	3.40	5.07	2.54	1.24	8.60	5.30	25.44	8.19	7.91	3.55

The analysis of EBV on DGV regression coefficients estimated for the validation dataset showed that models 1, 2, 3, and 4 maintained a tendency to underestimate breeding values, but the values of all the regression coefficients except MY decreased slightly. None of the linear models tested was clearly superior with regard to the studied traits.

Summing up, none of the linear models exhibited a significantly higher prediction accuracy in any of the comparisons between EBV and DGV, no matter whether correlations or regression were used: different types of design matrix for the random haplotype effects and the genetic variance equally or unequally divided into haplotypes had very little impact on the accuracy of prediction.

## Discussion

Interest in haplotypes as explanatory variables in linear models for predicting genomic breeding values was generated almost parallel to the possibility of using information derived from SNPs (Calus et al. 2008; Tzeng and Bondell 2010; Jiang et al. 2012; Cuyabano et al. 2014; Jónás et al. 2016; Hess et al. 2017). Haplotypes can be constructed using various methods based on, for example, the expectation-maximization algorithm (Excoffier and Montgomery 1995) or the Bayesian theory (Stephens et al. 2001). In this study, based on a previous investigation (Macierzyńska and Wierzbicki 2008), the latter method was used.

The number of haplotypes constructed for the purposes of this study depended inter alia on the size of blocks. The more SNPs in the block, the more potential allele configurations in the haplotype. Owing to the large number of haplotypes constructed (explanatory variables), among which there were many with a low probability of occurrence in the population, the criterion of their frequency (> 25%) in the population was used. A similar approach was used by Huang et al. (2007), who used the limiting value of this criterion computed as 1/*n* and 2/*n*, where *n* is the population size. Also, Kolbehdari et al. (2007) and Boleckova et al. (2012) showed that predictions of breeding values using haplotypes with higher frequencies were more accurate than using haplotypes with lower frequencies. In the study by Hayes et al. (2006), haplotypes with a frequency of less than 1% were eliminated. That frequency was much lower than the haplotype frequency used in the present study. This was because of the large number of haplotypes constructed and the limited number of individuals analyzed, which caused problems with the linear modeling. According to Calus et al. (2008), reducing the number of haplotypes may improve both the feasibility and the power of the model.

The correlations between EBV and DGV obtained for the test dataset were high and comparable with those reported by Szyda et al. (2009), who used linear models with SNPs as random explanatory variables. The correlations estimated for the validation dataset were much lower. This tendency was also indicated by Habier et al. (2007) and Moser et al. (2009). They reported that a higher accuracy of prediction was achieved for the validation dataset using models with random haplotype effects. Boleckova et al. (2012) drew a similar conclusion after having tested linear models with haplotypes as explanatory variables. A like tendency was described by Mucha and Wierzbicki (2012), who used simulated data in a haplotype-based breeding value prediction study. The correlations between EBV and DGV for production traits in the validation dataset were lower than the correlations presented by Solberg et al. (2008). The authors reported correlations from 0.69 to 0.86 using SNPs, and from 0.80 to 0.82 using haplotypes. Kolbehdari et al. (2007) and Calus et al. (2009) also reported higher correlations between EBV and DGV: they ranged from 0.72 to 0.85 and from 0.79 to 0.81, respectively. However, it should be noted that all these studies were carried out using simulated data.

According to Hess et al. (2017), who studied the accuracy of genomic selection in dairy cattle using fixed-length haplotypes, fitting covariates for fixed-length haplotype alleles rather than SNPs can increase the accuracy of genomic prediction by up to 5.5%. They also found that using shorter haploblocks led to a higher accuracy of prediction than using longer ones.

The data used in the present study were also used to predict the breeding values based on SNP effects (Szyda et al. 2011). The reference population in that study was a group of 984 bulls, 252 of which belonged to the validation set. The correlations between EBV and DGV were 0.38 for milk yield, 0.37 for protein yield, and 0.32 for fat yield. These values were lower than the correlations estimated using models with random haplotype effects described in the present study. Szyda et al. (2011) estimated different correlation coefficients (0.43 for milk yield, 0.44 for protein yield, 0.31 for fat yield) after having estimated the genomic enhanced breeding value (GEBV) by combining DGV and parental information (parent average). The correlations between GEBV and EBV for milk yield and protein yield were comparable to the correlations between DGV and EBV reported in the present study.

The regression coefficients of EBV on DGV for the validation dataset indicated underestimation of GBVs of the studied traits. This was particularly evident with regard to low heritability traits (NRK, PRP, OMC). The least biased predictors were obtained for STA, MY, PY, FY, and SCS. This confirms that more accurate predictors of breeding values are obtained for traits with higher heritability (Calus et al. 2008; Moser et al. 2010). In Ireland, genomic selection was also implemented with a relatively small reference population of 596 bulls, and a validation group of 207 bulls (Berry and Kearney 2009). EBV on DGV regression coefficients were at the level of 0.76 for milk yield, 0.78 for fat yield, 0.80 for protein yield, and 0.77 for somatic cell score, which suggests that the predictors of breeding values, despite the differences between the reference population sizes, were more accurate than the predictors obtained in this study. However, Berry and Kearney (2009) pointed out that the accuracy of the results could have been greater if information from the bull daughters from the validation dataset had been included in the test dataset.

An interesting proposal regarding genomic selection using haplotypes was given by Da (2015). That author developed a quantitative genetics-based multi-allelic haplotype model for integrating functional and structural genomic information using haplotypes separately or jointly with SNPs. This approach may be a significant contribution to improving the accuracy of genomic prediction.

In the present study, a generational partition of the dataset into two subsets to perform cross-validation was applied. However, it is known that the CV layouts may have an important effect on the accuracy of genomic predictions (Perez-Cabal et al. 2012; Gianola and Schön 2016). The results of the study reported by Pszczola et al. (2012) indicate that genetic relationships within and between training and testing datasets influence the reliability of direct genomic breeding values. A higher relationship of the evaluated animals to the reference population together with smaller average relationship within the reference population results in higher reliabilities of genomic predictions. Keeping this in mind, further studies using different cross-validation scenarios would be needed to draw more general conclusions from the results of the study presented.

In conclusion, the use of high-frequency haplotypes showed a tendency to underestimate DGVs. None of the models tested was clearly superior with regard to the traits studied. The type of design matrix for the haplotype effects (either containing the probabilities of a given haplotype being passed on by a particular bull or consisting of only ones, if a given bull had a specific haplotype, and of zeros if it did not) as well as the equal or unequal division of the genetic variance between haplotypes did not significantly affect the accuracy of prediction. DGVs of production and conformation traits as well as somatic cell score (medium or high heritability traits) were more accurate than those estimated for fertility traits (low heritability traits). The relatively low accuracy of genomic selection, especially for low heritability traits, could well have been higher if high-frequency haplotypes had been combined with SNPs alleles with known effects (Calus et al. 2008) or if haplotype effects had been combined with parental information (Szyda et al. 2011).
